# Crystal structure of the *Escherichia coli* transcription termination factor Rho

**DOI:** 10.1107/S2053230X20010572

**Published:** 2020-08-20

**Authors:** Chengcheng Fan, Douglas C. Rees

**Affiliations:** a Division of Chemistry and Chemical Engineering, California Institute of Technology, 1200 East California Boulevard, Pasadena, CA 91125, USA; b Howard Hughes Medical Institute, California Institute of Technology, 1200 East California Boulevard, Pasadena, CA 91125, USA

**Keywords:** membrane-protein purification, crystallization contaminant, transcription termination factor, cryoEM

## Abstract

During the crystal structure analysis of an ATP-binding cassette (ABC) transporter overexpressed in *Escherichia coli*, a contaminant protein was crystallized. The identity of the contaminant was revealed by mass spectrometry to be the *Escherichia coli* transcription terminator factor Rho, structures of which had been previously determined in different conformational states. Although Rho was present at only ∼1% of the target protein (a bacterial homolog of the eukaryotic ABC transporter of mitochondria from *Novosphingobium aromaticivorans*; *Na*Atm1), it preferentially crystallized in space group *C*2 as thin plates that diffracted to 3.30 Å resolution. The structure of Rho in this crystal form exhibits a hexameric open-ring staircase conformation with bound ATP; this characteristic structure was also observed on electron-microscopy grids of the *Na*Atm1 preparation.

## Abstract 2

A structure is reported of the *E. coli* transcription terminator factor Rho that was crystallized as an impurity present in preparations of an overexpressed bacterial membrane-transport protein.

## 1. Introduction   

One of the challenges in the crystallography of membrane proteins is their typically low expression level, which necessitates a significant degree of purification to separate the protein of interest from all other cellular proteins. This can consequently lead to the inadvertent purification of contaminant proteins that might otherwise be present at negligible levels when the target protein expresses at high levels. In unfortunate cases, these impurities may crystallize more readily than the target protein, leading to misplaced enthusiasm until the contaminant is recognized. As examples, the multi-drug efflux pump AcrB is a well known crystallization contaminant in membrane-protein preparations owing to its relatively high expression level during recombinant protein expressions with antibiotic selection and its nonspecific binding to Ni–NTA columns (Veesler *et al.*, 2008 ▸; Das *et al.*, 2007 ▸). Bacterioferritin has also been crystallized as a contaminant in preparations of cytochrome *cbb*_3_ oxidase (Nam *et al.*, 2010 ▸). In addition, exogenous proteins such as DNase, lysozyme and various proteases used in target protein purification have also been shown to be crystallization contaminants (Niedzialkowska *et al.*, 2016 ▸). Compilations facilitate the identification of crystals of contaminant proteins (Hungler *et al.*, 2016 ▸; Simpkin *et al.*, 2018 ▸), but the crystallization of ‘novel’ impurities is still a concern. In this work, we report the crystal structure of a previously unreported contaminating protein, the transcription termination factor Rho from *Escherichia coli*, which was obtained during the structural analysis of a bacterial ATP-binding cassette (ABC) transporter.

Rho is a hexameric RNA helicase that functions in transcription termination in *E. coli*. The six subunits together form a ring-like structure, and the structure switches between an open-ring staircase conformation and a closed-ring conformation coupled to the binding and hydrolysis of ATP (Skordalakes & Berger, 2003 ▸; Thomsen & Berger, 2009 ▸). Here, we present the crystal structure of Rho in an open-ring staircase conformation at 3.30 Å resolution with ATP bound.

## 2. Materials and methods   

### 2.1. Macromolecule production   

Rho copurified with the bacterial ABC exporter *Na*Atm1, which is a homolog of the ABC transporter of mitochondria (Atm1) from *Novosphingobium aromaticivorans*, using an *E. coli* expression system and a modified version of a previously published protocol (Lee *et al.*, 2014 ▸). Briefly, frozen *E. coli* cell pellets containing overexpressed *Na*Atm1 (and Rho) were lysed in lysis buffer consisting of 100 m*M* NaCl, 20 m*M* Tris pH 7.5, 40 m*M* imidazole pH 7.5, 10 m*M* MgCl_2_, 0.5%(*w*/*v*) *n*-dodecyl-β-d-maltopyranoside (DDM; Anatrace), 0.5%(*w*/*v*) octaethylene glycol monododecyl ether (C12E8; Anatrace) with the addition of lysozyme, DNase and protease inhibitor. After stirring for 3 h at 4°C, the lysate was subjected to ultracentrifugation at ∼113 000*g* for 45 min at 4°C. The supernatant was collected, loaded onto a pre-washed Ni–NTA column in buffer consisting of 100 m*M* NaCl, 20 m*M* Tris pH 7.5, 50 m*M* imidazole pH 7.5, 0.05% DDM, 0.05% C12E8 and eluted with the same buffer containing 350 m*M* imidazole pH 7.5. The protein was further purified by size-exclusion chromatography (SEC) using a HiLoad 16/60 Superdex 200 column (GE Healthcare) with SEC buffer consisting of 100 m*M* NaCl, 20 m*M* Tris pH 7.5, 0.05% DDM, 0.05% C12E8. Peak fractions were collected and concentrated to ∼20 mg ml^−1^ using a 100 kDa cutoff Amicon Ultra 15 concentrator (Millipore). Macromolecule-production information is summarized in Table 1 ▸.

### 2.2. Crystallization   

Rho crystallized during the crystallization trials of *Na*Atm1 under optimized conditions based on MemGold (Molecular Dimensions) condition No. 68 at 20°C by hanging-drop vapor diffusion. The final crystallization condition consisted of 100 m*M* NaCl, 100 m*M* Tris pH 8.3, 28% polyethylene glycol 2000 monomethyl ether (PEG 2000 MME), 0.2 *M* non­detergent sulfobetaine 221 (NDSB-221), 20 m*M* ATP pH 7.5. The crystallization sample was prepared in 1 m*M* ATP pH 7.5, 5 m*M* EDTA pH 7.5 in the presence and absence of 5 m*M* oxidized glutathione (GSSG) pH 7.5, which is a transport ligand for *Na*Atm1. Thin plate-shaped crystals appeared in about two weeks. The crystals were harvested in cryoprotectant solution consisting of 100 m*M* NaCl, 100 m*M* Tris pH 8.3, 28% PEG 2000 MME with PEG 400 at 10%, 15% and 20% before flash-cooling in liquid nitrogen. Crystallization information is summarized in Table 2 ▸.

### 2.3. Data collection and processing   

Crystals were screened on the GM/CA beamline 23-ID-B at the Advanced Photon Source (APS) and on beamline 12-2 at the Stanford Synchrotron Radiation Laboratory. The final data set was collected on the GM/CA beamline 23-ID-B with an EIGER 16M detector (Dectris) using *JBluIce*–*EPICS* (Stepanov *et al.*, 2011 ▸), processed and integrated with *XDS* (Kabsch, 2010 ▸) and scaled with *AIMLESS* (Evans & Murshudov, 2013 ▸). The crystals of Rho diffracted to about 3.30 Å resolution in space group *C*2, with unit-cell parameters *a* = 161.8, *b* = 101.9, *c* = 184.0 Å, β = 107.8°. Data-collection and processing statistics are summarized in Table 3 ▸.

### 2.4. Structure solution and refinement   

The self-rotation function was calculated with the *CCP*4 program *MOLREP* (Winn *et al.*, 2011 ▸). Molecular replacement was performed with *Phaser* in *Phenix* (Liebschner *et al.*, 2019 ▸) using a monomeric subunit of a previous structure of Rho with PDB code 1pvo (Skordalakes & Berger, 2003 ▸) as a model. Initial jelly-body refinements were carried out with *REFMAC*5 in *CCP*4 (Winn *et al.*, 2011 ▸). Subsequent iterative refinement and model-building runs were separately conducted with *phenix.refine* (Liebschner *et al.*, 2019 ▸) and *Coot* (Emsley *et al.*, 2010 ▸), respectively. The refined coordinates and structure factors have been deposited in the RCSB Protein Data Bank as entry 6wa8. Refinement statistics are summarized in Table 4 ▸.

### 2.5. Electron-microscopy sample preparation and data processing   

The expression plasmid for the membrane-scaffolding protein MSP1D1 was purchased from Addgene (plasmid No. 20061). The expression and purification of MSP1D1 were carried out using published protocols with minor modifications (Ritchie *et al.*, 2009 ▸). Reconstitution of *Na*Atm1 (and the Rho contaminant) with MSP1D1 was carried out with 1-palmitoyl-2-oleoyl-glycero-3-phosphocholine (POPC) at a 1:2:130 molar ratio of *Na*Atm1:MSP1D1:POPC. This reconstituted sample was incubated overnight at 4°C. After two hours of incubation, BioBeads were added at 200 mg ml^−1^ for detergent removal. The sample was then subjected to size-exclusion chromatography on a Superdex 200 Increase 10/300 column (GE Healthcare). The peak fractions were pooled and concentrated to ∼8 mg ml^−1^.

EM grids were prepared using a protein concentration of 4 mg ml^−1^ in the presence of 5 m*M* GSSG and 5 m*M* AMPPNP. 3 µl protein sample was applied onto freshly glow-discharged QuantiFoil Cu R2/2 300 mesh grids and blotted for 4 s with a blot force of 0 and 100% humidity at room temperature using a Vitrobot Mark IV (FEI). Data were collected on a 200 keV Talos Arctica with a Falcon III detector at a magnification of 92 000 and a total dose of 81 e Å^−2^ at the Caltech CryoEM Facility.

Data processing was performed with *cryoSPARC*2 (Punjani *et al.*, 2017 ▸), following motion correction with full-frame motion and estimation of the contrast transfer function (CTF) with *CTFFIND* (Rohou & Grigorieff, 2015 ▸). Particles were picked using a reconstruction of *Na*Atm1 as a template and extracted in *cryoSPARC*2. The initial 2D classification revealed a single class of Rho with ∼2500 particles. The particles were then 2D-classified again into five classes, with four good classes with a total of ∼2200 particles, as shown in Fig. 4(*b*).

## 3. Results and discussion   

**Figure 1:**
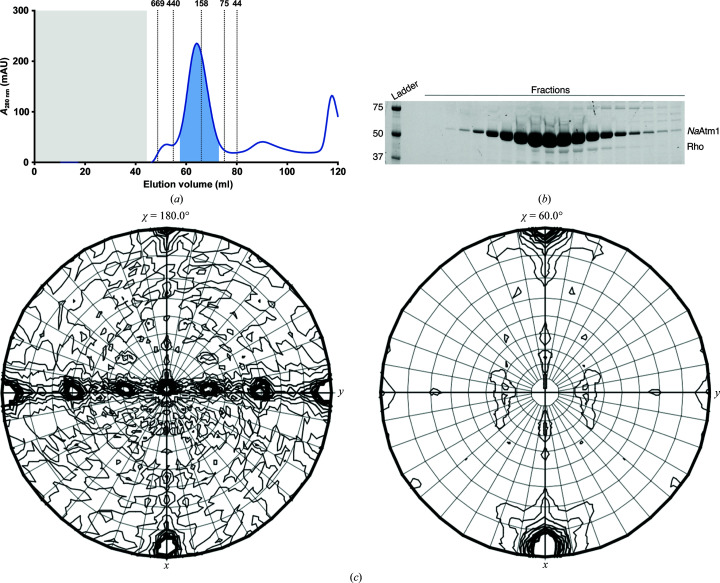
Purification and self-rotation function analysis of Rho. (*a*) Size-exclusion chromatograph of *Na*Atm1 purification using a HiLoad Superdex 200 16/60 column. The column void volume is colored gray, while the elution positions of various molecular-weight standards (in kDa) are marked on the chromatograph. (*b*) SDS–PAGE of the peak fractions of SEC purification from (*a*). (*c*) The χ = 180° and 60° sections of the self-rotation function calculated using *MOLREP* in *CCP*4 (Winn *et al.*, 2011 ▸) with an integration radius of 51 Å using diffraction data between 3.3 and 40 Å resolution.

Crystals of Rho were unexpectedly obtained during studies of the bacterial ABC transporter *Na*Atm1 from *N. aromaticivorans*. *Na*Atm1 was recombinantly expressed in *E. coli* BL21-Gold (DE3) cells with a C-terminal 6×His tag following a previously established protocol (Lee *et al.*, 2014 ▸). After solubilization of the *E. coli* cells in DDM and C12E8, protein purification proceeded by Ni–NTA affinity purification followed by size-exclusion chromatography (Fig. 1 ▸ *a*). SDS–PAGE gels indicated a high degree of purity, although in subsequent analysis of overloaded gels a small amount of another protein was present at a molecular weight of ∼40 kDa (Fig. 1 ▸ *b*).

The crystals obtained during the crystallization optimization belonged to space group *C*2, with unit-cell parameters *a* = 161.8, *b* = 101.9, *c* = 184.0 Å, β = 107.8°. The asymmetric unit volume was of sufficient size to accommodate an *Na*Atm1 dimer (molecular weight of 133 kDa). Analysis of the self-rotation function calculated from the diffraction data revealed a noncrystallographic symmetry (NCS) sixfold axis offset ∼3–4° from the crystallographic *a* axis. Interaction of the perpendicular twofold and sixfold axes generates a set of non­crystallographic twofold rotation operations separated by 60° in the plane perpendicular to the NCS sixfold axis (Fig. 1 ▸ *c*). Given the unit-cell dimensions, this apparent NCS was incompatible with dimeric *Na*Atm1, which raised the possibility that a contaminant had crystallized. With an estimated molecular weight of ∼40 kDa based on the observation of a faint impurity band in the gel and a Matthews coefficient analysis, we performed molecular replacements with known crystallization contaminants (Hungler *et al.*, 2016 ▸; Simpkin *et al.*, 2018 ▸), which all failed to yield a molecular-replacement solution.

**Figure 2:**
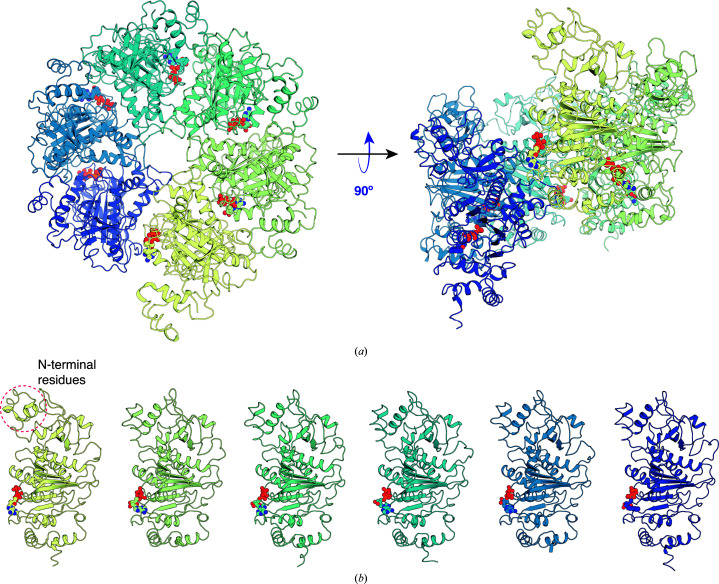
Crystal structure of Rho. (*a*) Overall view of Rho in broken-staircase conformation with ATP bound. (*b*) Single subunit of Rho with ATP bound. The six subunits are shown separately and ATP is shown as red spheres. The dashed circle identifies the N-terminal ∼50 residues of the Rho subunit positioned at the break in the hexameric staircase arrangement; this region has rearranged in this subunit relative to the conformation exhibited in the other five subunits.

The identification of Rho (molecular weight 47 kDa) was established by a mass-spectrometric analysis of the peptides prepared by trypsin digestion of the protein in the SDS–PAGE bands. Using this information, we were able to obtain a molecular-replacement solution using Rho in the AMPPNP-bound state (PDB entry 1pvo; Skordalakes & Berger, 2003 ▸) as the input model. The molecular-replacement results established that in these crystals Rho adopts a six-membered broken-staircase conformation. The structure was refined to an *R*_work_ and *R*_free_ of 25.2% and 29.6%, respectively (Table 4 ▸). The electron-density map also revealed ATP to be bound in all six subunits (Fig. 2 ▸ *a*).

**Figure 3:**
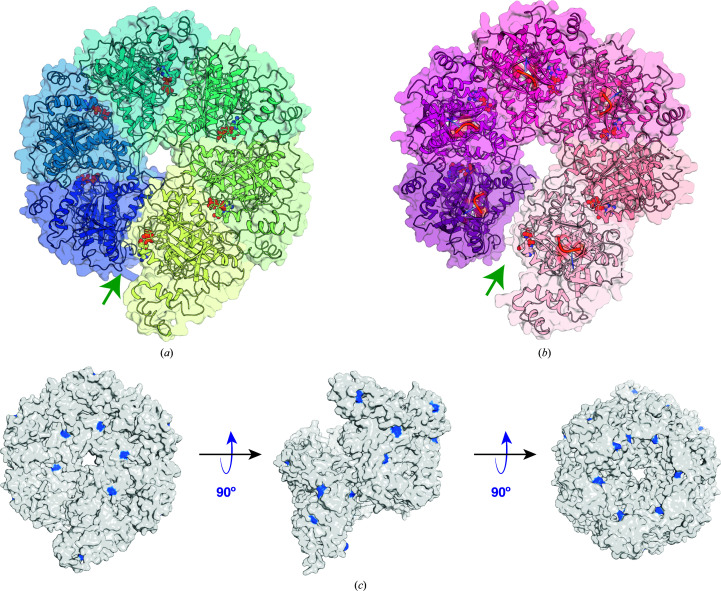
Overall architecture of Rho. Overall structural representations of (*a*) ATP-bound Rho (this study) and (*b*) the AMPPNP-bound structure of Rho (PDB entry 1pvo; Skordalakes & Berger, 2003 ▸). (*c*) Distribution of surface histidine residues (blue) in the ATP-bound structure of Rho (this work). The spacings between the surface-exposed histidines are several nanometres and are comparable to the loading density of His-tagged proteins bound to Ni–NTA beads (Hayworth & Hermanson, 2014 ▸), which presumably allows multiple binding sites to Ni–NTA and contributes to the observed affinity of Rho for Ni–NTA resin.

The individual Rho subunits are structurally similar overall, except for the terminal subunit at the ‘break’ in the staircase, where the first 50 residues at the N-terminus exhibited a shift of 2–8 Å relative to the other monomers (Fig. 2 ▸ *b*). The r.m.s.d. between the present structure and the molecular-replacement input model (PDB entry 1pvo) is 2.5 Å using the C^α^ positions for the superposition and 3.3 Å when using all atoms of the six subunits in the hexamer. The r.m.s.d.s between individual subunits in the present structure and PDB entry 1pvo are 0.59–0.73 Å, reflecting their similar tertiary structures. The relationship between adjacent subunits in the broken staircase of the present structure may be approximated by a screw operation with a rotation around the screw axis of 60.5° per subunit and a corresonding translation along the axis of −7.7 Å. In comparison, the corresponding values for PDB entry 1pvo are 57.9° and −8.2 Å, respectively. Reflecting the larger rotation angle per subunit, the present structure exhibits a more closed ring in comparison to the original broken-staircase structure (Figs. 3 ▸ *a* and 3 ▸ *b*). At the level of subunit–subunit interactions, however, the differences are subtle and we could not identify the specific interactions responsible for these differences in hexamer structure. Of note, the nucleotide-binding site is at the interface between subunits and small changes in subunit–subunit interactions may reflect the presence of different nucleotides: ATP in this structure and either no nucleotide (apo), ATPγS or AMPPNP in other open-ring structures (Skordalakes *et al.*, 2005 ▸; Skordalakes & Berger, 2003 ▸).

How did Rho end up in our crystallization conditions? Our hypothesis is that during the overexpression of proteins the *E. coli* transcription and translation machineries are highly expressed for the production of mRNAs and recombinant proteins, respectively. Rho, as the termination factor, would plausibly be overexpressed as part of the transcription-termination process; thus, it is likely that Rho is a general contaminant and does not arise specifically from the overexpression of *Na*Atm1. The fact that Rho eluted from the Ni–NTA column along with His-tagged *Na*Atm1 suggests that there is nonspecific binding to the Ni^2+^ resin by the histidine residues distributed throughout the whole protein (Fig. 3 ▸ *c*). The open-ring conformation that Rho adopts in solution (Thomsen *et al.*, 2016 ▸) with a molecular weight of 282 kDa for the hexamer apparently has a comparable hydrodynamic radius to *Na*Atm1, with a dimer molecular weight of 133 kDa in addition to the detergent micelle. Given the low abundance, the presence of Rho in the SEC fractions was only detected in hindsight. Also in hindsight, Rho was not present in the original *Na*Atm1 purification, which included a membrane-isolation step in which Rho was presumably removed (Lee *et al.*, 2014 ▸); in the present work the membrane-isolation step was omitted and Rho subsequently copurified with *Na*Atm1.

**Figure 4:**
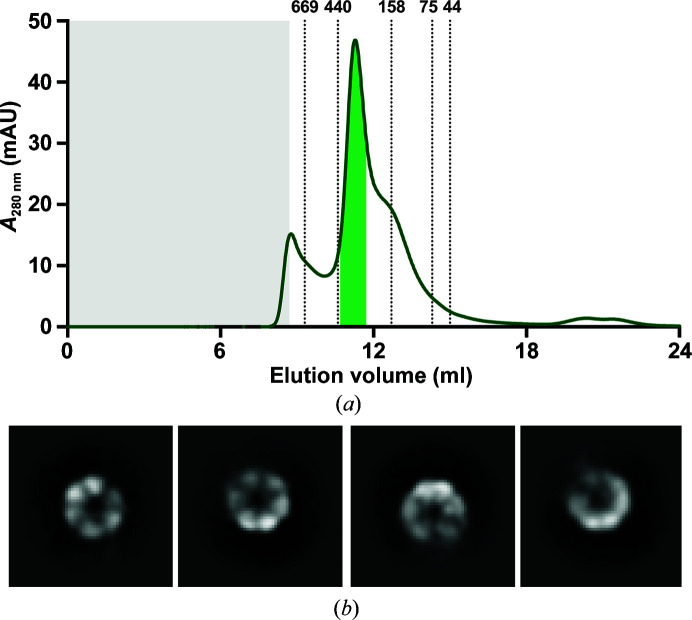
Single-particle cryo-EM analysis of *Na*Atm1 samples containing Rho. (*a*) Size-exclusion chromatograph of the reconstituted *Na*Atm1 in MSP1D1 nanodiscs using a Superdex 200 Increase 10/300 column. The column void volume is colored gray, while the elution positions of various molecular-weight standards (in kDa) are marked on the chromatograph. (*b*) 2D classes of Rho illustrating the hexameric arrangement with a diameter of ∼100 Å. The box size of the 2D classes is 284 × 284 Å.

We have also observed Rho in single-particle cryoEM studies of *Na*Atm1 reconstituted in membrane-scaffolding protein (MSP) nanodiscs. The *Na*Atm1 nanodisc sample was prepared by incubating detergent-purified *Na*Atm1 with MSPs and lipids, and was further purified by size-exclusion chromatography. Similar to the purification in detergent, the peak fractions were collected for single-particle cryoEM sample preparation (Fig. 4 ▸ *a*). The 2D classification reported one class of Rho in the broken hexameric state (Fig. 4 ▸ *b*), again suggesting that Rho has a similar hydrodynamic radius and plausibly a similar molecular weight to our reconstituted *Na*Atm1 in nanodiscs.

In a structural analysis of a prokaryotic chloride channel, a single peptide of Rho was identified in a mass-spectrometric analysis of the gel band (Abeyrathne & Grigorieff, 2017 ▸), representing the first time, to our knowledge, that Rho has been identified as a possible contaminant during membrane-protein expression. As demonstrated in this report, Rho can crystallize even in the presence of a large excess of other proteins, and thus it should be added to the list of known contaminant proteins in crystallography.

## Supplementary Material

PDB reference: *E. coli* transcription termination factor Rho, 6wa8

## Figures and Tables

**Table 1:** Macromolecule-production information The protein was expressed in *E. coli* from the native promotor without the use of an expression plasmid.

Source organism	*E. coli*
DNA source	Genomic DNA from *E. coli*
Expression host	*E. coli* BL21-Gold (DE3)
Complete amino-acid sequence of the construct produced	MNLTELKNTPVSELITLGENMGLENLARMRKQDIIFAILKQHAKSGEDIFGDGVLEILQDGFGFLRSADSSYLAGPDDIYVSPSQIRRFNLRTGDTISGKIRPPKEGERYFALLKVNEVNFDKPENARNKILFENLTPLHANSRLRMERGNGSTEDLTARVLDLASPIGRGQRGLIVAPPKAGKTMLLQNIAQSIAYNHPDCVLMVLLIDERPEEVTEMQRLVKGEVVASTFDEPASRHVQVAEMVIEKAKRLVEHKKDVIILLDSITRLARAYNTVVPASGKVLTGGVDANALHRPKRFFGAARNVEEGGSLTIIATALIDTGSKMDEVIYEEFKGTGNMELHLSRKIAEKRVFPAIDYNRSGTRKEELLTTQEELQKMWILRKIIHPMGEIDAMEFLINKLAMTKTNDDFFEMMKRS

**Table 2:** Crystallization

Method	Hanging-drop vapor diffusion
Plate type	Hampton Research VDX
Temperature (K)	293
Protein concentration (mg ml^−1^)	<1
Buffer composition of protein solution	100 m*M* NaCl, 20 m*M* Tris pH 7.5, 1 m*M* ATP
Composition of reservoir solution	100 m*M* NaCl, 100 m*M* Tris pH 8.3, 20 m*M* ATP, 200 m*M* NDSB-221, 28% PEG 2000 MME
Volume and ratio of drop	1 µl protein solution + 1 µl reservoir solution
Volume of reservoir (µl)	500

**Table 3:** Data collection and processing Values in parentheses are for the outer shell.

Diffraction source	GM/CA 23-ID-B, APS
Wavelength (Å)	0.9793
Temperature (K)	100
Detector	EIGER 16M
Crystal-to-detector distance (mm)	400
Rotation range per image (°)	0.2
Total rotation range (°)	360
Exposure time per image (s)	0.2
Space group	*C*2
*a*, *b*, *c* (Å)	161.75, 101.90, 184.02
α, β, γ (°)	90, 107.8, 90
Mosaicity (°)	0.11
Resolution range (Å)	39.79–3.30 (3.42–3.30)
Total No. of reflections	304610 (29798)
No. of unique reflections	43031 (4465)
Completeness (%)	99.9 (99.7)
Multiplicity	7.1 (6.7)
〈*I*/σ(*I*)〉	6.6 (1.5)†
*R*_p.i.m._	0.103 (0.729)
Overall *B* factor from Wilson plot (Å^2^)	79.1‡

† Overall resolution cutoff determined by data completeness and CC_1/2_ > 0.50 in the high-resolution shell. *I*/σ(*I*) falls below 2.0 past 3.46 Å resolution.

‡ There were ice rings in the data.

**Table 4:** Structure solution and refinement Values in parentheses are for the outer shell.

Resolution range (Å)	38.51–3.30 (3.42–3.30)
Completeness (%)	99.6 (97.3)
σ Cutoff	8.9 (1.3)
No. of reflections, working set	40807 (3928)
No. of reflections, test set	2095 (217)
Final *R*_cryst_	0.252 (0.320)
Final *R*_free_	0.296 (0.364)
No. of non-H atoms	
Total	19776
Protein	19590
Ligand	186
R.m.s. deviations	
Bond lengths (Å)	0.002
Angles (°)	0.58
Average *B* factors (Å^2^)	
Overall	101.8
Protein	101.4
Ligand	143.2
Ramachandran plot	
Most favored (%)	95.5
Allowed (%)	4.2
PDB entry	6wa8
